# Electromagnetic Properties of Carbon Gels

**DOI:** 10.3390/ma12244143

**Published:** 2019-12-10

**Authors:** Jimena Castro-Gutiérrez, Edita Palaimiene, Jan Macutkevic, Juras Banys, Polina Kuzhir, Sébastien Schaefer, Vanessa Fierro, Alain Celzard

**Affiliations:** 1 Institut Jean Lamour, Université de Lorraine, CNRS, 88000 Épinal, France; jimena.castro-gutierrez@univ-lorraine.fr (J.C.-G.); sebastien.schaefer@univ-lorraine.fr (S.S.); vanessa.fierro@univ-lorraine.fr (V.F.); 2 Faculty of Physics, Vilnius University, Sauletekio av. 9, 01100 Vilnius, Lithuania; edita.palaimiene@ff.vu.lt (E.P.); juras.banys@ff.vu.lt (J.B.); 3 Centre for Physical Science and Technology, Sauletekio av. 3, 01100 Vilnius, Lithuania; jan.macutkevic@gmail.com; 4 Institute of Photonics, University of Eastern Finland, Yliopistokatu 7, 80101 Joensuu, Finland; polina.kuzhir@gmail.com; 5 Institute for Nuclear Problems, Belarusian State University, 220030 Minsk, Belarus

**Keywords:** carbon gel, electromagnetic properties, electrical conductivity, glasslike carbon

## Abstract

The electromagnetic properties of various carbon gels, produced with different bulk densities, were investigated in a wide frequency range (20 Hz–36 GHz). The values of dielectric permittivity and electrical conductivity at 129 Hz were found to be very high, i.e., more than 10^5^ and close to 100 S/m, respectively. Both strongly decreased with frequency but remained high in the microwave frequency range (close to 10 and about 0.1 S/m, respectively, at 30 GHz). Moreover, the dielectric permittivity and the electrical conductivity strongly increased with the bulk density of the materials, according to power laws at low frequency. However, the maximum of microwave absorption was observed at lower densities. The DC conductivity slightly decreased on cooling, according to the Arrhenius law. The lower activation energies are typical of carbon gels presenting lower DC electrical conductivities, due to a higher number of defects. High and thermally stable electromagnetic properties of carbon gels, together with other unique properties of these materials, such as lightness and chemical inertness, open possibilities for producing new electromagnetic coatings.

## 1. Introduction

Carbon gels have received great attention because of their applications in water purification [1], electrochemical energy storage [2], catalysis [3] and gas separation [4]. These properties and applications are related to their developed surface areas, allowing for excellent adsorption properties [5]. The details of their preparation, corresponding structures and main physicochemical properties have been reviewed in a recent monograph [6]. In brief, carbon gels are produced by pyrolysis of thermoset polymer gels, which are themselves prepared by polycondensation of organic monomers diluted in a solvent. Once the crosslinking is achieved, the solvent can be removed by various processes, namely subcritical drying, supercritical drying or freeze-drying. These different routes lead to xerogels, aerogels and cryogels, respectively. Xerogels generally present the lowest porosity and the lowest surface areas, due to the action of capillary forces during solvent evaporation. This induces a significant shrinkage and hence a narrowing and even a collapse of the porosity. In contrast, aerogels are those in which the porosity is most preserved, precisely because of the absence of capillary forces during drying, leading to the highest pore volumes and the highest surface areas. Finally, cryogels have intermediate porosities and surface areas, because of the crystallisation of the solvent, which usually produces a porosity that is more preserved than that of xerogels but also coarser than that of aerogels. More information about synthesis, structure, properties and industrial applications can be found elsewhere [7].

When the gels have a phenolic nature, they are infusible and present an excellent carbon yield, of about 45%–50%, when submitted to pyrolysis at temperatures of at least 1173 K. Consequently, the gels lose weight and shrink but in a homogeneous and homothetic way. They are therefore converted into carbon gels while retaining their original texture, based on spherical nodules [5,6,7] and the references therein. Mesopores (range of size 2–50 nm) correspond to the voids between the carbon nodules, whereas micropores (<2 nm) are created during pyrolysis and located within the nodules. Therefore, carbon gels are porous monoliths with a hierarchical, fully open porous structure. Obviously, their carbonaceous character also gives them a significant electrical conductivity, which mainly depends on the thermal history, on the nature of the precursor and on the total porosity.

The electromagnetic properties of porous carbon structures are an important topic in the field of electromagnetic interference suppression [8]. It is commonly accepted that carbon foams are good candidates for electromagnetic shielding materials [9,10,11]. Various other porous carbon structures, as well as carbon-filled composites, are also often investigated for electromagnetic applications [12,13,14]. However, the electromagnetic properties of carbon gels are still relatively unknown. Moreover, for porous carbon materials, it is very important to find relationships between the porous carbon structure, their macroscopic parameters (such as bulk density) and broadband electromagnetic properties over a wide temperature range [9].

The purpose of the present work is to investigate the broadband electromagnetic properties of various types of carbon gels and their dependence on bulk density and pore size. Because this work deals with a first evaluation of electromagnetic properties of carbon gels, by which their properties need to be primarily measured and understood, no finalised application can be suggested at this stage, but it opens the route to a future optimisation of these materials.

## 2. Materials and Methods

### 2.1. Preparation of Carbon Gels

Carbon gels have been prepared according to a procedure published elsewhere [15] and very slightly modified herein. In brief, resorcinol (R), formaldehyde (F) and sodium carbonate (C) were dissolved in distilled water (W) using a molar ratio R/F of 0.5, a dilution molar ratio D = W/(R + F + C) of either 5.7 or 20 and a molar ratio R/C of either 50, 500 or 1000. These three parameters are indeed known to have a strong influence on the porous structure of phenolic gels [15] and the references therein.

Gelation was then performed in 15 mm diameter sealed test tubes at 358 K for three days, and then, the tubes were broken to recover the gels. The latter were next submitted to drying in different ways. Xerogels were obtained after five days of vacuum drying at 333 K, followed by heating at 423 K in vacuum. Cryogels were obtained by first exchanging the solvent entrapped in the fresh gels with *tert*-butanol at 323 K for three days, during which *tert*-butanol content was replaced daily, followed by freezing at 173 K and vacuum drying for five days. Finally, aerogels were produced by exchanging the solvent entrapped in the fresh gels with dry ethanol for three days, replacing it every 24 h, then using an automatic critical point drier (TOUSIMIS Autosamdri 815-A, Rockville, MD, USA) by which the ethanol was replaced by CO_2_ in supercritical conditions. More details about these processes have been presented elsewhere [16,17,18,19].

Finally, once dried, the gels were pyrolysed in a flow of pure argon at 2 K/min up to the final temperature, 1323 K, which was kept for 45 min before the furnace was allowed to cool down under argon flow. The samples were called Cx-y—where C stands for “carbon”, x is the resorcinol/carbonate molar ratio and y is the dilution ratio—followed by the type of material obtained—either XERO, CRYO or AERO, depending on the drying route.

### 2.2. Characterisation of Carbon Gels

The porous texture of carbon gels has been investigated, as explained in the work where the synthesis protocol was described [15]. Given that the syntheses are highly reproducible, the corresponding characterisation has not been repeated, and the porous texture parameters were directly taken from the same paper, especially the skeletal density and the maximum pore diameter. The skeletal density, *ρ_s_*, represents the density of the (non-porous) carbon phase alone, i.e., the carbon backbone, and it was measured by helium pycnometry. The maximum pore diameter, *d_max_*, corresponds to the limit under which smaller pores represent 95% of the total pore volume; it was deduced for each sample from the pore size distributions obtained by nitrogen adsorption at 77 K, using the Broekhoff–de Boer method. We selected *d_max_* instead of any other parameter related to pore size, because it appeared in ref. [15] as the most relevant to discuss the effect of synthesis conditions on the final porosity of the materials. Moreover, it was the parameter most closely related to the R/C ratio and the total pore volume, and it allowed us to build a phase diagram elucidating the mechanical properties of carbon gels in relation to their porous structure. We thus decided to follow the same method in the discussion of the results.

Interested readers can also refer to ref. [15] for electron microscopy images of these materials. We have decided not to show them again here, because all these carbon gels present the typical nodular structure expected for materials derived from resorcinol–formaldehyde resins gelled in diluted conditions. Moreover, as highlighted in ref. [15], all samples observed by scanning electron microscopy present the same irregular surface and look similar at this magnification. Indeed, the pores are too small to be observed by such technique.

Only the bulk density was remeasured herein, due to its sensibility to little differences in the drying process. The measurement was carried out according to the envelope method, using a Geopyc 1360 apparatus (Micromeritics) and an ultrafine powder (Dryflo^®^) with a liquid-like flow behaviour. In the experiment, the powder was forced to conform perfectly to the contours of the sample of known weight, without penetrating or compressing it, which was made possible thanks to a position sensor in the holder of calibrated volume to measure the volume occupied by the material, whatever its geometry. Based on the bulk density, *ρ_b_*, and the skeletal density, *ρ_s_*, the total porosity *Φ* can be calculated as *Φ* = 1 − *ρ_b_*/*ρ_s_*.

Dielectric permittivity and electrical conductivity were measured at low frequency, i.e., between 20 Hz and 1 MHz, using a Hewlett-Packard (Palo Alto, CA, USA) 4284A LCR-meter. The measurements were based on the use of equivalent circuits to determine the capacitance and the loss tangent. From these quantities, the complex dielectric permittivity ε* = *ε′* – i*ε″* was calculated according to planar capacitor formulas. The electrical conductivity was calculated as *σ* = *ε″ε_0_ω*.

The electromagnetic properties of the carbon gels were also investigated in the microwave frequency range (26–38 GHz, Ka-band) using a 7.2 × 3.4 mm^2^ rectangular cross-section waveguide. An Elmika (Vilnius, Lithuania) 2400 scalar network analyser was used to measure the scalar scattering parameters. Rod-like samples with a diameter of about 1 mm were investigated and placed at the centre of the waveguide with their axis parallel to the electric field vector. All samples were glued with silver paint to the sample holder. The complex dielectric permittivity was calculated with a modified Newton optimisation algorithm based on the microwave theory formalism (see ref. [20] for details).

## 3. Results and Discussion

### Porosity of Carbon Gels

**Figure 1:**
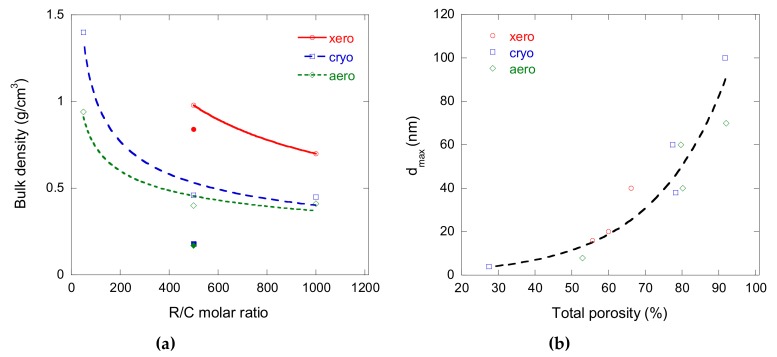
(**a**) Changes of bulk density as a function of the resorcinol to sodium carbonate molar ratio (R/C), depending on the drying mode. The empty symbols and full symbols refer to dilution ratios of 5.7 and 20, respectively, and the curves are just guides for the eye between points corresponding to a dilution ratio of 5.7 only; (**b**) Maximum pore diameter as a function of total porosity for all carbon gels of the present study.

The main porous texture parameters of the carbon gels can be found in Table 1. It can be seen that more sodium carbonate produced narrower pores. It is indeed well known with this kind of gel that a higher pH induces a decrease in the diameter of the carbon nodules and therefore in the width of the mesopores between them (see, for instance, ref. [21] and the references therein). It can also be observed that increasing the dilution ratio with a constant amount of sodium carbonate had the opposite effect, which was also expected, given that more diluted gels contain more solvent and therefore lead to larger pores after drying. Another logical trend is that xerogels have much narrower pores and much lower porosities than aerogels and cryogels, all other things being equal. Finally, with a constant sodium carbonate and dilution ratio, aerogels were slightly more porous than cryogels, but their values of *d_max_* were rather similar. A summary of these trends is presented in Figure 1.

**Figure 2:**
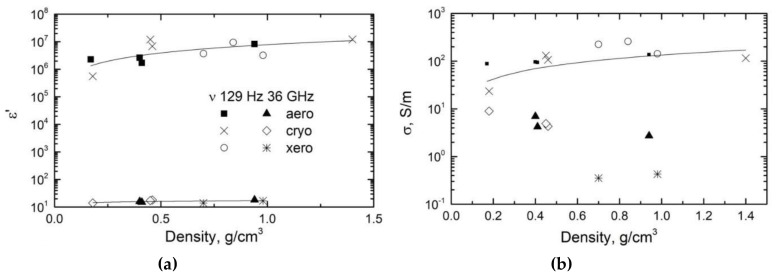
(**a**) Dielectric permittivity and (**b**) electrical conductivity of various carbon gels at different frequencies as a function of bulk density.

The dielectric permittivity and the electrical conductivity of various carbon gels as a function of bulk density at different frequencies are presented in Figure 2. At low frequency, the values were very high (at 0.4 g/cm^3^ and 129 Hz (an arbitrarily chosen low value of frequency), *ε’* > 10^6^ and *σ* ≈ 100 S/m) and increased with the bulk density. In the microwave frequency range, these quantities were much lower (at 0.4 g/cm^3^ again and 30 GHz, *ε’* ≈ 18 and *σ* ≈ 4 S/m), but the dielectric permittivity also tended to increase. The microwave dielectric properties were better than those of wormhole-like mesoporous carbon [22] but lower than those of carbon foams [9]. Power laws describe the results at low frequency very well:

*ε’ ~ k ρ_b_^s^*, (1)

*σ ~ l ρ_b_^t^*, (2)

where *ρ_b_* is again the bulk density of carbon gels, *k* and *l* are proportionality constants and *t* and *s* are parameters that depend on the geometry of the conductive phase (i.e., the carbon gel backbone). It should be recalled here that Equations (1) and (2) apply in microwaves, as proven for instance in ref. [23] and in many other papers. Nevertheless, in the microwave frequency range, the dependence of electrical conductivity as a function of density is very scattered, so that only the dependence of dielectric permittivity as a function of density was fitted with Equation (1). The scattering at microwaves can be due to the different types of carbon gels and measurements errors.

The fitting parameters obtained from the dependences of dielectric permittivity and electrical conductivity on bulk density are listed in Table 2. The value of *t* for the conductivity at 129 Hz was lower than that predicted by the percolation theory for any three-dimensional (3D) medium (1.6–2) or even for a two-dimensional (2D) medium (1.1–1.33); therefore, it cannot be related to a lower dimensionality [24]. Its non-universality can be explained by the Swiss cheese model (also called random void model), which considers spherical voids of uniform size randomly placed in a uniform transport medium [25]. Indeed, pores in carbon gels can be seen as spherical voids. However, in our opinion, the relatively low value of *t* is more likely to be related to the slow increase of electrical conductivity due to the fact that carbon gel densities were rather high, i.e., very far from the percolation threshold. Moreover, the values of *s* and *t* were close to each other according to percolation universality [26], and the value of *s* decreased with frequency as expected for percolative systems [23]. The dielectric permittivity at microwaves was slightly lower than the one reported for carbon foams, while the values of the microwave electrical conductivities of carbon foams and carbon gels were very similar [9,27].

When plotted as a function of maximum pore size (not shown), *ε’* and *ε”* slightly decreased when *d_max_* increased, which is a logical behaviour given that bulk density (and hence porosity) and pore size are directly related to each other, as suggested by Figure 1b. Indeed, it should be recalled here that the wavelength was much larger than any pore size considered here, so that any observed trend as a function of *d_max_* was expected to be due to related changes of *ρ_b_* [9,28,29]. Moreover, the variations of *ε’* and *ε”* were very limited with respect to the considered range of values of *d_max_*, and therefore, it is more than likely that neither *ε’* and *ε”* really depend on pore size. The same was already observed with various kinds of carbon foams with independent variations of pore size and porosity and for which clear conclusions could thus be drawn: whereas the pore size was far higher than the values given in Table 1 and hence more likely to interact with microwaves, the only trends in electromagnetic properties were observed as a function of porosity, not of pore size [9,27].

**Figure 3:**
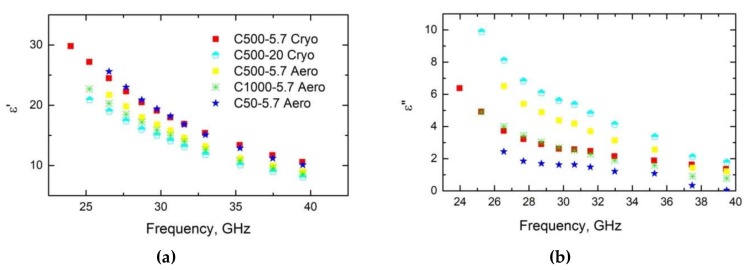
Dielectric permittivity and dielectric losses of various carbon gels in the microwave frequency range.

Figure 3 shows the frequency dependencies of the dielectric permittivity *ε’* and the dielectric losses *ε”* for carbon gels in the microwave frequency range. The dielectric permittivity was higher than the dielectric losses, indicating that *ε’* = *ε”* at lower frequencies. Moreover, both *ε’* and *ε”* increased with carbon gel density but decreased with frequency. The latter is typical of Maxwell–Wagner relaxation at higher frequencies, at which *ε” > ε’* [30].

**Figure 4:**
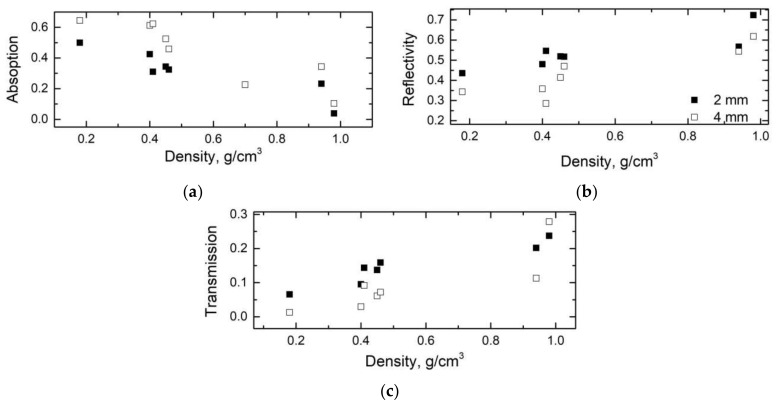
(**a**) Absorption, (**b**) reflection and (**c**) transmission of carbon gels of different thicknesses calculated at 30 GHz.

From the data of dielectric permittivity at 30 GHz, the electromagnetic shielding of different layers of carbon gels has been calculated. The results are presented in Figure 4, based on calculations using the equations presented in ref. [31]. It can be concluded that layers of carbon gel with a thickness of 2 or 4 mm and a low density of about 0.2 g/cm^3^ presented an absorption close to 0.5. At a higher density, the absorption decreased—especially at about 1 g/cm^3^, where it was only 0.1, on average, compensated by a much higher reflectivity, close to 0.7. The maximum of absorption at low densities, i.e., at a higher porosity, was related to a higher electrical conductivity (and dielectric losses) (see again Figure 2), and this can be explained by microwaves scattering at pores, grain boundaries and interfaces [28]. A similar maximum has been observed for carbon foams [9].

**Figure 5:**
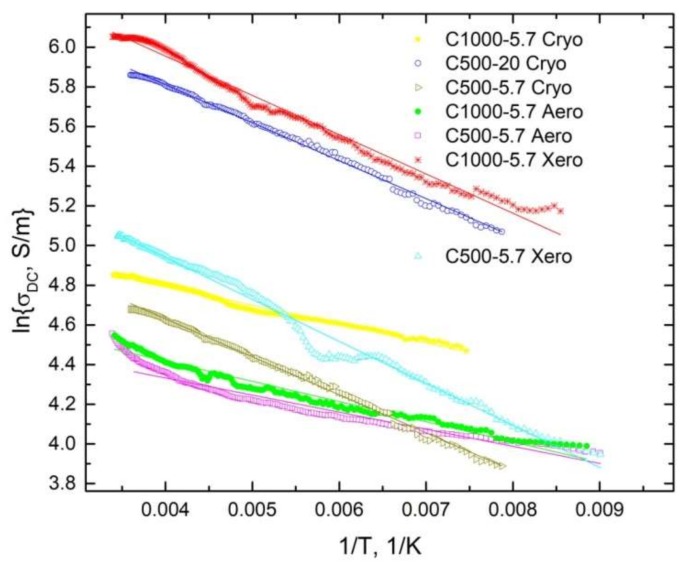
Temperature dependence of the DC conductivity of various carbon gels.

**Figure 6:**
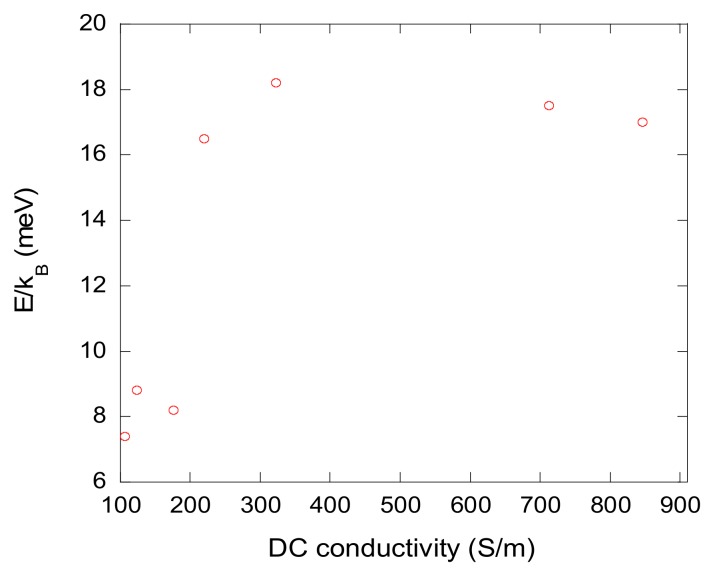
Activation energy derived from the application of Equation (3) to the data of Figure 5, as a function of the DC electrical conductivity.

The temperature dependence of the DC conductivity of the carbon gels is presented in Figure 5. All trends can be well described by Arrhenius’s law:

(3) $$\sigma_{DC}=\sigma_{0}e^{-\frac{E}{kT}},$$

where *σ_0_* is the pre-exponential factor and *E* is the activation energy. The values of activation are given in Table 3, and their dependence on DC conductivity is presented in Figure 6. The activation energy clearly increased with the DC conductivity. This can be explained by the higher concentration of defects in the least conductive carbon gels; hence, the electrical transport through these defects required a lower activation energy.

## 4. Conclusions

The electromagnetic properties of various carbon gels, produced at different densities, have been studied over a wide range of frequencies (20 Hz–36 GHz). The values of dielectric permittivity and electrical conductivity at 129 Hz were very high, i.e., more than 10^5^ and nearly 100 S/m, respectively. Both parameters decreased sharply with frequency, as is typically the case for Maxwell–Wagner relaxation, but remained significant in the microwave frequency range (at 30 GHz, the dielectric permittivity was close to 10, and the electrical conductivity was about 0.1 S/m). Moreover, these two quantities strongly increased with the density according to power laws at low frequency. However, the maximum of microwave absorption was observed at lower densities, which is related to microwaves scattering at pores, grain boundaries and interfaces. No influence of pore size was noticed, in agreement with former studies dealing with electromagnetic properties of other porous carbons. The DC conductivity decreased slightly on cooling according to the Arrhenius law. The lower activation energies are typical of carbon gels with lower electrical conductivities, due to the probable presence of defects. To conclude, being very lightweight, chemically inert and, at the same time, highly conductive in a wide frequency range (including microwaves), carbon gels are valuable materials for electromagnetic shielding applications.

## Figures and Tables

**Table 1:** Main pore texture parameters of the carbon gels.

Sample	Bulk Density, *ρ_b_* (g/cm^3^)	Skeletal Density, *ρ*_s_ (g/cm^3^)	Maximum Pore Size, *d_max_* (nm)	Total Porosity, *Φ* (%)
C1000-5.7 XERO	0.70	2.07	40	66.2
C500-5.7 XERO	0.98	2.21	16	55.7
C500-20 XERO	0.84	2.10	20	60.0
C1000-5.7 CRYO	0.45	2.00	60	77.5
C500-5.7 CRYO	0.46	2.12	38	78.3
C50-5.7 CRYO	1.40	1.93	4	27.5
C500-20 CRYO	0.18	2.19	100	91.8
C1000-5.7 AERO	0.41	2.02	60	79.7
C500-5.7 AERO	0.40	2.03	40	80.3
C50-5.7 AERO	0.94	2.00	8	53.0
C500-20 AERO	0.17	2.16	70	92.1

**Table 2:** Parameters corresponding to the fits of Equations (1) and (2) to the data of Figure 2.

*k* or *l*	*t* or *s*	Comment
15.87	0.99	*ε’* at 129 Hz
4.9	0.716	*σ* at 129 Hz
2.84	0.094	*ε’* at 30 GHz

**Table 3:** Fitting parameters of the data of DC conductivity versus temperature.

Sample	*σ_0_*, S/m	*E*/*k_B_*, K (meV)
C1000-5.7 AERO	124	102 (8.8)
C500-5.7 AERO	107	86 (7.4)
C1000-5.7 CRYO	176	95 (8.2)
C500-20 CRYO	713	191 (17.5)
C1000-5.7 XERO	846	197 (17.0)
C500-5.7 XERO	323	211 (18.2)
C500-5.7 CRYO	220	191 (16.5)
